# ^99m^Tc-sodium phytate is a valid alternative to the gold-standard ^99m^Tc-sulfur colloid in the measurement of gastric emptying among healthy multi-ethnic Asian population: results of a randomized cross-over trial

**DOI:** 10.1186/s12876-020-01426-5

**Published:** 2020-08-31

**Authors:** Norazlina Mat Nawi, Nashrulhaq Tagiling, Mohd Fazrin Mohd Rohani, Wan Mohd Nazlee Wan Zainon, Muhammad Saifuddin Zanial, Mung Seong Wong, Yeong Yeh Lee

**Affiliations:** 1 Department of Nuclear Medicine, Radiotherapy and Oncology, School of Medical Sciences, Universiti Sains Malaysia, Kubang Kerian, 16150 Kelantan Malaysia; 2 Hospital USM, Universiti Sains Malaysia, Kubang Kerian, 16150 Kelantan Malaysia; 3 Nuclear Medicine Department, Hospital Kuala Lumpur, Kuala Lumpur, 50586 Malaysia; 4 School of Dental Sciences, Universiti Sains Malaysia, Kubang Kerian, 16150 Kelantan Malaysia; 5 Department of Medicine, Hospital Raja Perempuan Zainab II, Kota Bharu, 15586 Kelantan Malaysia; 6 Department of Medicine, School of Medical Sciences, Universiti Sains Malaysia, Kubang Kerian, 16150 Kelantan Malaysia

**Keywords:** Gastric emptying, Reference values, Single-photon emission-computed tomography, Radiopharmaceuticals, Sodium Phytate, Sulfur colloid

## Abstract

**Background:**

It is unclear if the ^99m^Tc-sodium phytate (^99m^Tc-SP) is as reliable as the gold-standard ^99m^Tc-sulfur colloid (^99m^Tc-SC) for gastric emptying scintigraphy (GES). This study is aimed to compare the emptying rates of both radiotracers in a prospective, randomized cross-over trial and to determine the normative data of a healthy multi-ethnic Asian population.

**Methods:**

Out of the 44 healthy individuals screened, 31 (14 females; mean age: 28.4 ± 7.0 years) were enrolled and underwent GES using the standardized egg-white meal. All participants were randomly assigned to either ^99m^Tc-SP or ^99m^Tc-SC on the first GES session before crossed over to the other formulation after 2 weeks.

**Results:**

Both kits achieved the radiochemical purities of > 95%. The median rate (95th upper normative limit) of gastric emptying, reported as total gastric meal retention between ^99m^Tc-SP and ^99m^Tc-SC, was found to be comparable at all measured time points: 0.5 h [85.0% (96.6%) vs. 82.0% (94.0%)], 1 h [70.0% (86.4%) vs. 65.0% (86.6%)], 2 h [31.0% (55.8%) vs. 25.0% (64.4%)], 3 h [7.0% (26.3%) vs. 5.0% (29.9%)], and 4 h [3.0% (10.3%) vs. 2.0% (9.9%)]; *P* > 0.05. In addition, both radiotracers correlated well (Kendall’s Tau (τ) coefficient = 0.498, *P* < 0.001) and presented with a good agreement at the 4th-hour time frame based on the Bland-Altman plot analysis.

**Conclusions:**

^99m^Tc-SP is a valid radiotracer alternative to ^99m^Tc-SC for routine GES examination. The normative values for both radiotracers have also been determined for the healthy multi-ethnic Asian population.

**Trial registration:**

This trial was registered retrospectively in the Thai Clinical Trials Registry on May 23rd, 2020 (Identifier: TCTR20200526004; http://www.clinicaltrials.in.th/index.php?tp=regtrials&menu=trialsearch&smenu=fulltext&task=search&task2=view1&id=6296).

## Background

Gastric emptying scintigraphy (GES), up to the present time, remains the gold-standard test for gastroparesis and functional dyspepsia [1]. The modus operandi of GES is a non-invasive tool to quantify the emptying of a physiologic caloric meal from the stomach into the small bowel [2, 3]. Due to its reliability, GES has become the benchmark for other diagnostic tests such as isotope breath test, magnetic resonance imaging, antro-duodenal manometry, and motility capsule [3, 4].

Since its inception in the 1960s, there have been many protocols designed to optimize GES; however, no definitive standard has yet to emerge despite the continuous efforts by various nuclear medicine societies [5, 6]. In a recently published 2020 guideline for clinical radiopharmaceuticals by the Administration of Radioactive Substances Advisory Committee (ARSAC), there are two chemical forms of technetium 99-metastable (^99m^Tc) based radiotracers that are eligible for gastrointestinal (GI) motility studies: colloids and non-absorbable compounds [7]. Despite such specification, there is a considerably wide selection of radiotracers that belong to the colloid category. ^99m^Tc-sulfur colloid (^99m^Tc-SC), for example, is known as the gold-standard colloidal radiotracer for GES. It is considered as the gold-standard as recommended by the GI Council of the Society of Nuclear Medicine and Molecular Imaging (SNMMI), the American Neurogastroenterology and Motility Society (ANMS), as well as the approval by the United States’ Food and Drug Administration (FDA) agency [5, 8]. However, the widespread use of ^99m^Tc-SC in low- and middle-income countries (LMICs) has been hindered due to its expensive price tag.

It is, therefore, attractive to have an alternative option in these countries. It is also important to note there is still a limited amount of literature on other radiotracers, and to the best of our knowledge, in vivo studies on the comparison with ^99m^Tc-SC is non-existent. In the meantime, perhaps one of the more affordable and commercially-available radiocolloids is the ^99m^Tc-sodium phytate (^99m^Tc-SP). ^99m^Tc-SP is well recognized in diagnostic imaging as a radiopharmaceutical product for liver-spleen tract examination. Studies have advocated ^99m^Tc-SP as a suitable colloidal radiotracer for GES because it possesses good labeling stability in vitro (92% at 3 h) within an egg-based meal [9]. Generally, ^99m^Tc-SP has been used by only several centers including in Thailand and Brazil, both of which are categorized as LMICs [10, 11].

Based on the discussion above, we aimed to objectively determine the reliability of ^99m^Tc-SP versus ^99m^Tc-SC for the measurement of gastric emptying rates and to define the normative values for a healthy multi-ethnic Asian population.

## Methods

The study protocol has received approval from the institution’s Human Research Ethics Committee (Reference number: USM/JEPeM/15070248), following the Declaration of Helsinki. The trial was registered in the Thai Clinical Trials Registry system (Identifier: TCTR20200526004; Website: http://www.clinicaltrials.in.th). Also, this report was written in adherence to the Consolidated Standards of Reporting Trials (CONSORT) 2010 guidelines.

### Study participants

Prospective participants were invited to join the study using word of mouth and advertisement. Healthy participants were screened and consecutively recruited within the campus compound (Health Campus, Universiti Sains Malaysia, Kelantan, Malaysia). All participants, aged 18 years old and above, were required to provide written informed consent before participating in the study. The exclusion criteria included those with an egg allergy, past and current history of chronic medical, psychiatric, and surgical illnesses and those currently on medication(s), which might affect the GI function.

Using a single-center, randomized cross-over trial design, each participant underwent two GES sessions with an interval period of 2 weeks. Such an approach was made to eliminate any possibility of carryover effect and to avoid significant changes in the participants’ body weight or metabolic parameters. Simple randomization between ^99m^Tc-SC and ^99m^Tc-SP was done by an internal medicine physician (MSZ). The participants were randomized in a 1:1 allocation, based on a randomization table generated by Microsoft® Excel (Microsoft Corp., USA) to ensure that either ^99m^Tc-SC or ^99m^Tc-SP is received in the first session. Subsequently, they are crossed over to the other radiotracer formulation in the second session.

### Scintigraphy procedure

Quality control for both kits of ^99m^Tc-SC (Pharmalucence, Inc., USA) and ^99m^Tc-SP (Technephyte; Center of Molecular Research, Russia) was first performed using the Tec-Control™ Chromatography strips (Biodex Medical Systems, USA). The radiochemical analysis results showed that both radiotracers possessed similar high purity values (99.7%) indicative of excellent labeling efficiency and desirability for in vivo diagnostic testing [12].

All participants were instructed to fast for at least 4–6 h and withhold any tobacco use for at least 24 h. Upon arrival to the imaging suite, they were given a standardized low-fat solid meal comprised of 118 ml (60 kcal) of egg-white mixed with either ^99m^Tc-SC or ^99m^Tc-SP of 37 MBq (1 mCi). The egg-white was cooked to a scrambled consistency on a non-stick skillet and served with two slices of white bread (120 kcal), 30 g strawberry jam (75 kcal), and 120 ml water to provide bulk [5, 6]. Duration for meal ingestion was capped at 15 min but optimally within 10 min.

GES was performed using a dual-head, large field-of-view single-photon emission-computed tomography/computed tomography scanner (Discovery™ SPECT/CT NM 670 Pro; GE Healthcare, USA) with a low-energy high-resolution collimator. A small ^99m^Tc radiolabeled marker of 0.37 MBq (10 μCi) was placed over the xiphisternum throughout the entire GES examination for repositioning purposes and region-of-interest (ROI) drawing. Static anterior and posterior images of 128 × 128 matrix were then taken simultaneously for 60 s each at standard time points (0, 0.5, 1, 2, 3, 4 h) after meal ingestion in the upright position. The energy window was centered on the photopeak for ^99m^Tc (140 keV), with a window width of 20%. Motion correction was applied to the acquired images, and the gastric ROI was manually delineated on the Xeleris™ 3.1 Workstation (GE Healthcare, USA). A nuclear medicine physician (NMN) and a medical physicist (NT) were assigned to analyze the images obtained jointly.

### Data and statistical analysis

The acquired data included the decay-corrected geometric mean of the anterior and posterior gastric counts for each time point. They were expressed as percentages of radioactivity remaining inside the whole stomach (total gastric meal retention or GMR – primary outcome). The total gastric counts at time zero (immediate GES) were normalized to 100%. Subsequently, a linearly-fitted time-activity profile for gastric emptying was generated based on the GMR datasets for each radiotracer to determine the solid-phase half-emptying time (T_1/2_ – secondary outcome).

The data analyses were conducted using JMP® Pro 13 (SAS Institute Inc., USA) and OriginPro 2019b (OriginLab Corp., USA) software. The descriptive data for GMRs and T_1/2_ were expressed in the form of upper normative limit values (90th and 95th percentile), median, interquartile range (IQR), mean, standard deviation (SD), and the mean’s 95% confidence interval (CI). The reported normative values for GMRs and T_1/2_ were generated from 1000 bootstrap replicates of the smoothed empirical likelihood quantile dataset, and its values were equipped with the estimated 90% CIs following the recommendation of the Clinical & Laboratory Standards Institute (CLSI) [13]. The probability of normality was evaluated using the Shapiro-Wilk test, while time-related differences in GMR and T_1/2_ between both radiotracers were assessed using the Wilcoxon’s Signed-Rank test for matched-pairs. Furthermore, the changes in the participants were measured during the cross-over trial regardless of the GES sequence (period effect) and performed non-parametrically using the Mann-Whitney *U* test.

A Bland–Altman (B-A) plot analysis was conducted to evaluate the intra-subject variation of GMRs in respective groups of ^99m^Tc-SC and ^99m^Tc-SP and to determine the agreement between both radiotracers. To keep it simple, we chose to analyze the 4th-hour GMR dataset only because of its high sensitivity to detect gastric dysfunction [1, 14, 15]. We also decided to adopt the modified criteria of the upper normative limit for the 4th-hour time frame (normal gastric emptying < 16% GMR versus abnormal gastric emptying ≥16% GMR), as stratified by DiBaise et al. [5, 16]. For a clinically meaningful difference, the maximum allowable mean differences in GMR value between the two radiotracers at the 4th-hour was set to no more than ±6%. The methodological agreement was defined as upper and lower 95% line of agreement (LoA) limits falling within the maximum allowable difference. Overall, *P*-value < 0.05 is considered as statistically significant.

As for sample size, no formal calculations were undertaken due to the exploratory nature of this study and lack of previous data (no clinical trial has evaluated the feasibility and reliability of ^99m^Tc-SP against ^99m^Tc-SC on GES). Therefore, we followed a general rule-of-thumb, whereby a minimum of 12 participants per trial arm was regarded as adequate to conduct a 2-period cross-over pilot study [17].

## Results

**Fig. 1:**
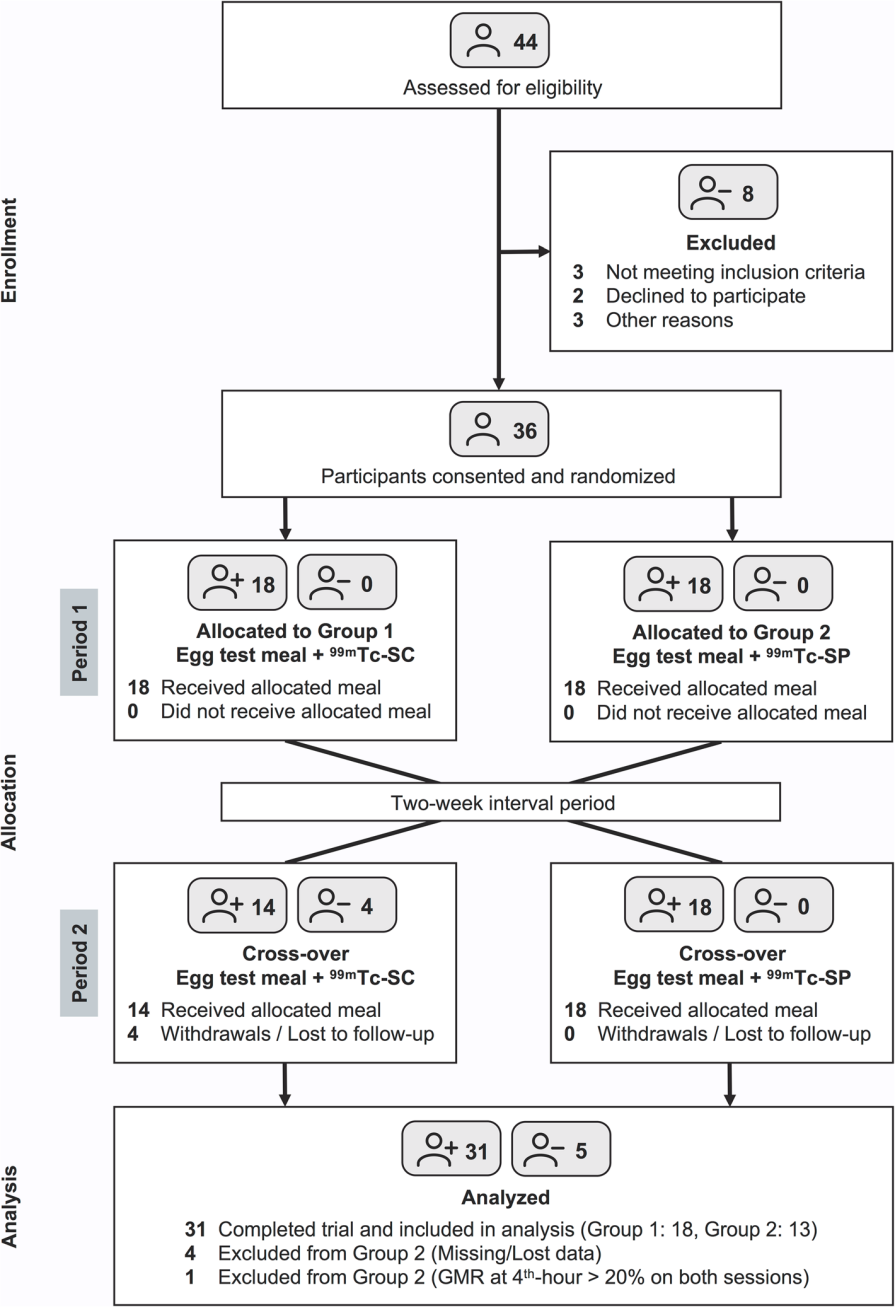
Study flowchart for the randomized cross-over trial

Out of the 44 healthy individuals screened, 31 were selected as participants of this study and were included in the final analysis (14 females; mean age: 28.4 ± 7.0 years), while the rest were excluded from the study due to medical reasons, presence of GI symptoms, and withdrawals (Fig. 1). About two-thirds of the studied population are from the Bumiputra group, which is made up of native Malays and other indigenous ethnicities in Malaysia (71.0%; 22/31), followed by Chinese (16.1%; 5/31) and Indians (12.9%; 4/31). The participants’ overall mean weight is 65.8 ± 14.1 kg (range: 40–101 kg) and the overall mean body mass index (BMI) is 24.5 ± 4.3 kg/m^2^ (range: 17.1–35.3 kg/m^2^). The first trial arm (Group 1: ^99m^Tc-SC, then ^99m^Tc-SP) consisted of 18 participants while the second trial arm (Group 2: ^99m^Tc-SP, then ^99m^Tc-SC) comprised of 13 participants. At the baseline, there are no significant differences in sex and BMI between the two randomized groups (Table 1; *P* > 0.05).

**Table 1:** Baseline participants’ characteristics between groups

Variables		Overall population	Group 1	Group 2	Statistics
Age (years)		28.4 (7.0)	25.9 (5.9)	32.4 (7.0)	*P* = 0.025^a^
Sex (*n*)	Male (%)	17 (54.8%)	7 (44.4%)	10 (76.9%)	*P* = 0.067^b^
	Female (%)	14 (45.2%)	11 (55.6%)	3 (23.1%)	
Weight (kg)		65.8 (14.1)	63.8 (14.1)	68.6 (14.2)	*P* = 0.378^a^
Height (m)		1.64 (0.1)	1.59 (0.1)	1.69 (0.1)	*P* = 0.005^a^
BMI (kg/m^2^)		24.5 (4.3)	24.9 (4.5)	23.9 (4.2)	*P* = 0.535^a^

Data are presented as mean (SD), except for sex

Group 1: ^99m^Tc-SC, then ^99m^Tc-SP (*n* = 18)

Group 2: ^99m^Tc-SP, then ^99m^Tc-SC (*n* = 13)

^a^Mann-Whitney *U* test

^b^Fisher Exact test

**Fig. 2:**
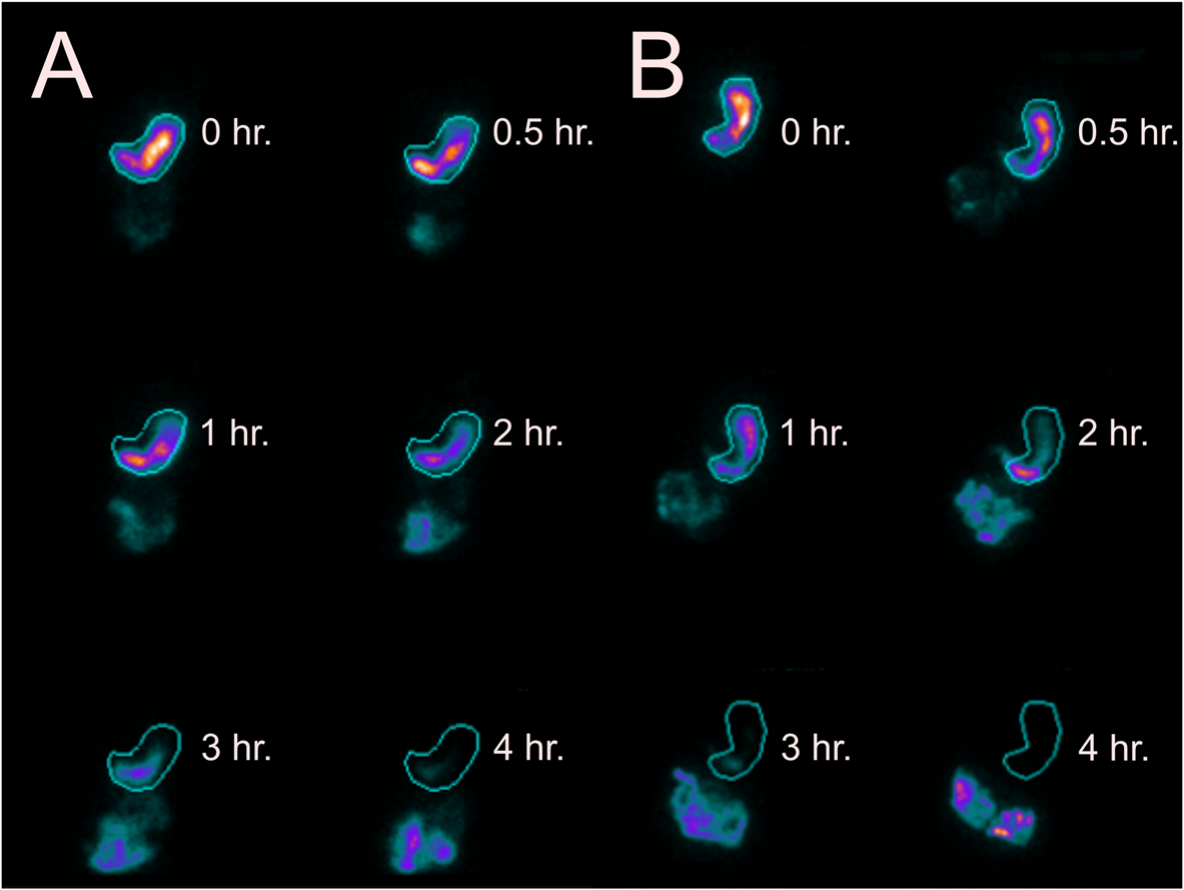
Paired anterior images of the 4-h gastric emptying scintigraphy protocol acquired from a healthy female participant. (**a**) ^99m^Tc-SC, and (**b**) ^99m^Tc-SP

**Fig. 3:**
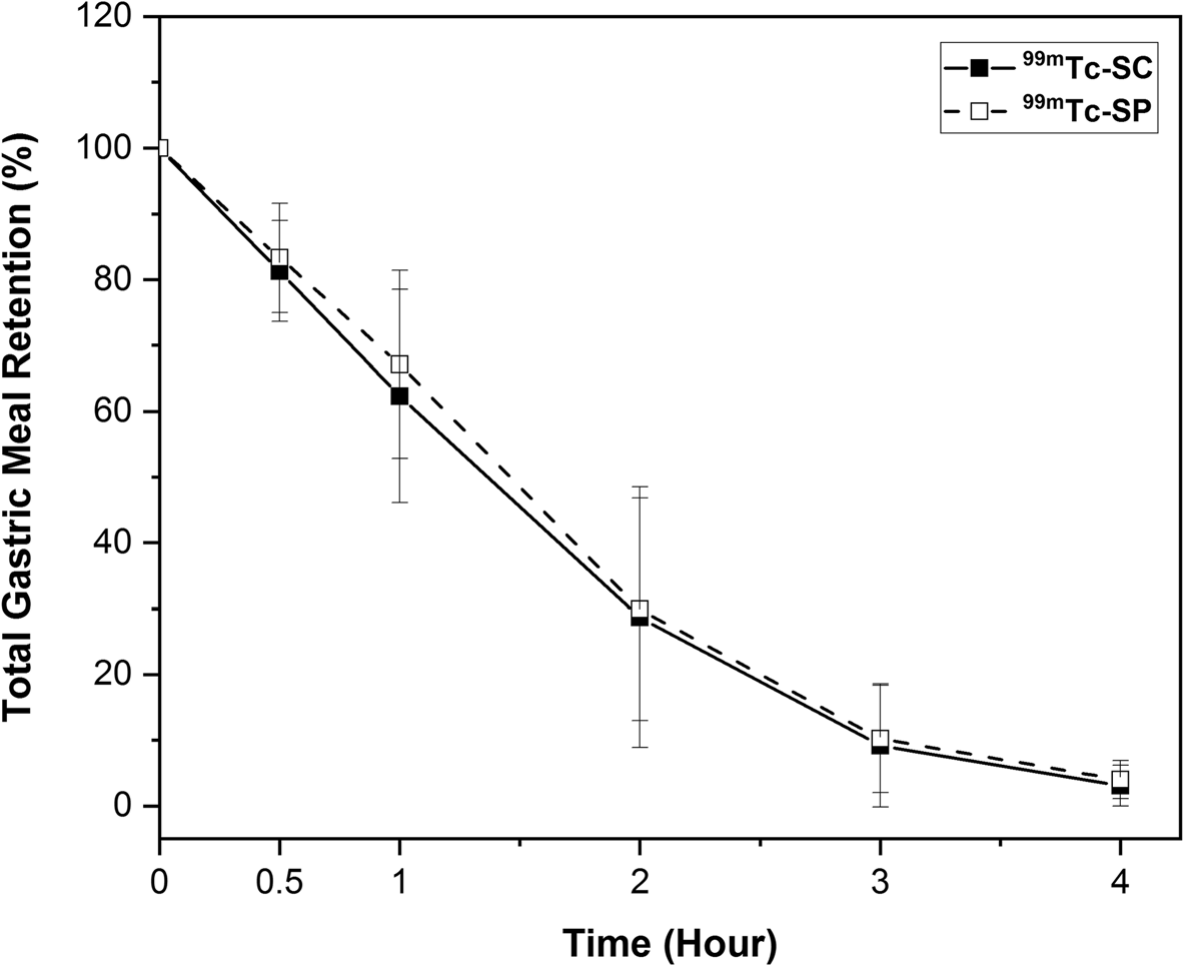
Gastric emptying profile for the 4-h standardized egg-white solid-meal labeled with ^99m^Tc-SC (solid black line) and ^99m^Tc-SP (dashed black line)

**Fig. 4:**
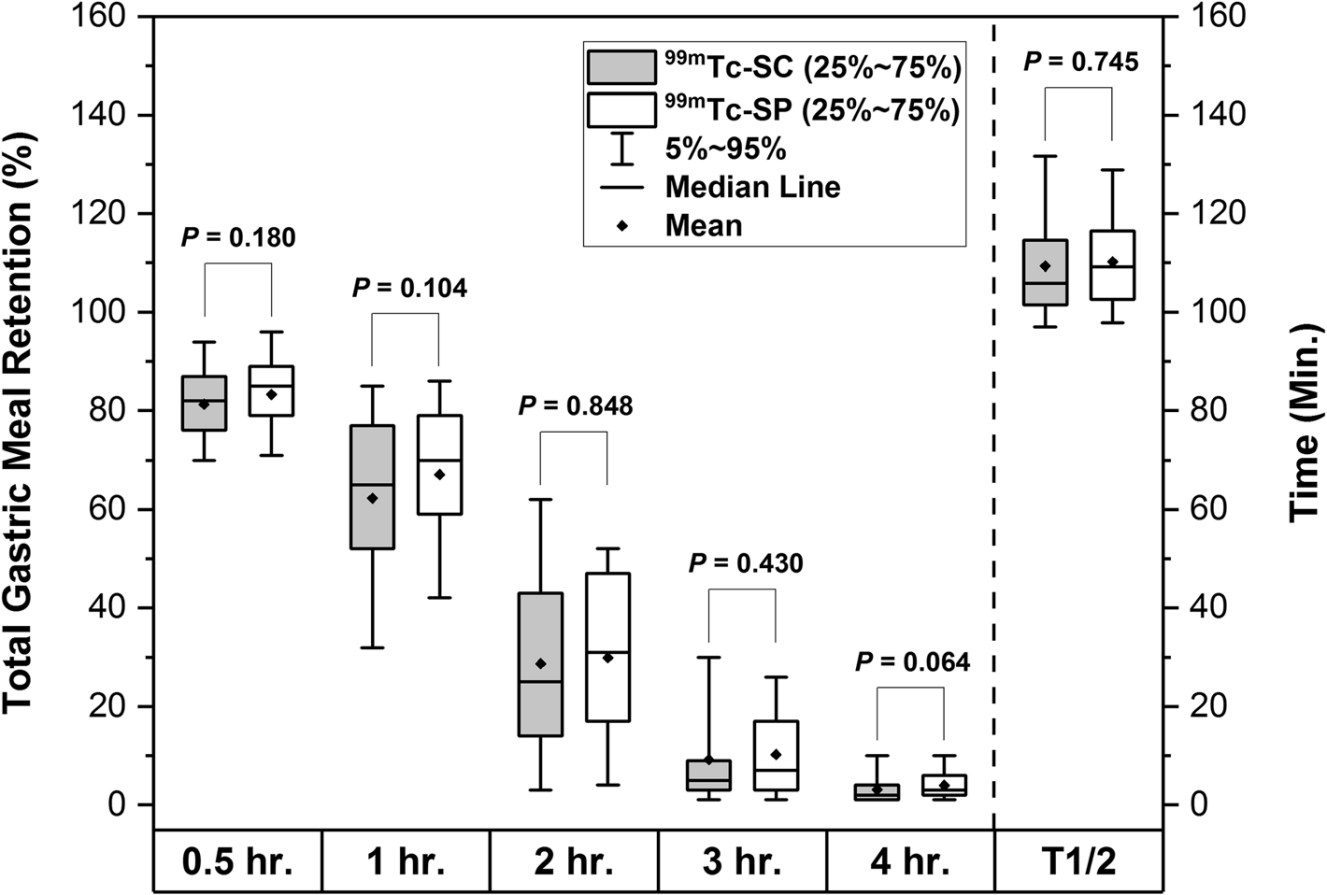
Box-and-whiskers plot for total gastric meal retention (GMR) and gastric emptying half time (T_1/2_) for standardized egg-white solid-meal labeled with ^99m^Tc-SC (grey box) and ^99m^Tc-SP (white box). As indicated in the brackets, there are no significant differences in GMR and T_1/2_ between both radiotracers (*P* > 0.05)

No activity was observed in the GI tract, except for the labeled bolus. It was also reported that all participants showed no signs of vomiting or partial ingestion of the test meal during the examination process. Figure 2 demonstrates one of the 62 paired GES images considered for analysis. The descriptive data of GMRs for ^99m^Tc-SC and ^99m^Tc-SP labeled meals are shown in Table 2. In brief, we found no statistically significant differences in time-related GMRs and linearly-fitted T_1/2_ between ^99m^Tc-SC and ^99m^Tc-SP (all *P* > 0.05). Figure 3 shows the comparison of the time-activity gastric emptying profile generated using the entire dataset of 31 participants (mean ± SD) for both radiotracers, while Fig. 4 shows its corresponding box-and-whiskers plot. The change in gastric emptying parameters between the sessions for Group 1 and Group 2 was summarized in Table 3. Statistical analysis on the cross-over design revealed no evidence of period effect across all of the investigated parameters (*P* > 0.05).

**Table 2:** Descriptive statistics of gastric emptying scintigraphy for standardized egg-white solid-meal labeled with ^99m^Tc-SC and ^99m^Tc-SP

Radiotracer	Parameters		Normative values^**a**^		Median (IQR)	Mean (SD)	95% CI of Mean	Normality
			90th percentile (90% CI)	95th percentile (90% CI)				
^99m^Tc-SC	Total gastric meal retention	0.5 h. (%)	91.7 (88.9–94.3)	94.0 (91.0–96.2)	82.0 (11.0)	81.3 (7.7)	78.5–84.1	*P* = 0.891
		1 h. (%)	83.2 (78.9–86.9)	86.6 (82.8–90.2)	65.0 (25.0)	62.3 (16.2)	56.3–68.2	*P* = 0.251
		2 h. (%)	60.2 (48.8–64.8)	64.4 (57.9–69.1)	25.0 (29.0)	28.7 (19.8)	21.4–36.0	*P* = 0.019^b^
		3 h. (%)	26.9 (15.9–30.8)	29.9 (25.4–33.5)	5.0 (6.0)	9.2 (9.4)	5.7–12.7	*P* < 0.001^b^
		4 h. (%)^c^	7.4 (5.0–11.2)	9.9 (6.4–14.0)	2.0 (3.0)	3.1 (3.1)	2.0–4.2	*P* < 0.001^b^
	Linear-fit Solid T_1/2_	T_1/2_ (min.)	127.2 (121.3–131.5)	130.5 (124.9–134.0)	105.9 (13.1)	109.4 (10.8)	105.4–113.3	*P* < 0.001^b^
^99m^Tc-SP	Total gastric meal retention	0.5 h. (%)	94.1 (90.8–96.9)	96.6 (93.2–99.0)	85.0 (10.0)	83.3 (8.3)	80.3–86.3	*P* = 0.385
		1 h. (%)	83.8 (80.3–86.3)	86.4 (82.8–89.1)	70.0 (20.0)	67.1 (14.3)	61.9–72.3	*P* = 0.063
		2 h. (%)	52.4 (47.8–55.2)	55.8 (51.4–58.8)	31.0 (30.0)	29.9 (16.9)	23.7–36.1	*P* = 0.038^b^
		3 h. (%)	21.6 (17.8–27.5)	26.3 (20.6–31.1)	7.0 (14.0)	10.2 (8.2)	7.2–13.2	*P* = 0.015^b^
		4 h. (%)^c^	8.8 (6.5–10.5)	10.3 (7.9–11.3)	3.0 (4.0)	4.0 (2.9)	2.9–5.1	*P* < 0.001^b^
	Linear-fit Solid T_1/2_	T_1/2_ (min.)	123.7 (119.1–130.0)	128.4 (122.1–132.9)	109.2 (13.9)	110.2 (9.6)	106.7–113.7	*P* = 0.084

^a^Determined based on bootstrapped-estimates of smoothed empirical likelihood quantiles (bootstrap samples = 1000)

^b^Non-normal distribution (*P* < 0.05)

^c^GMR percentage at the 4th-hour was modified according to DiBaise’s stratification: normal < 16%, abnormal ≥16% [5, 16]

**Table 3:** Comparison of gastric emptying parameter outcomes based on test meal sequence

Parameters		Sequence	Session		Within-individual difference: ^**99m**^Tc-SC - ^**99m**^Tc-SP
			1	2	
Total gastric meal retention	0.5 h. (%)	**Group 1**	83.3 (6.0)	84.1 (6.8)	−0.8 (6.5)
		**Group 2**	82.2 (10.3)	78.5 (9.0)	−3.6 (12.8)
		**Period effect**	–	–	−2.0 (9.5); 95% CI: −5.5, 1.5
					*P* = 0.325
	1 h. (%)	**Group 1**	68.6 (11.4)	71.4 (10.1)	−2.9 (10.1)
		**Group 2**	61.1 (17.2)	53.6 (18.3)	−7.5 (24.5)
		**Period effect**	–	–	−4.8 (17.4); 95% CI: −11.2, 1.6
					*P* = 0.458
	2 h. (%)	**Group 1**	36.7 (18.3)	34.7 (15.5)	2.0 (11.3)
		**Group 2**	23.2 (17.0)	17.6 (16.7)	−5.6 (22.4)
		**Period effect**	–	–	−1.2 (17.0); 95% CI: −7.4, 5.0
					*P* = 0.155
	3 h. (%)	**Group 1**	12.6 (11.1)	12.3 (9.0)	0.3 (7.4)
		**Group 2**	7.3 (6.2)	4.5 (2.5)	−2.9 (6.8)
		**Period effect**	–	–	−1.0 (7.2); 95% CI: −3.7, 1.6
					*P* = 0.446
	4 h. (%)	**Group 1**	4.2 (3.7)	4.7 (3.2)	−0.5 (2.1)
		**Group 2**	2.9 (2.3)	1.5 (0.9)	−1.4 (2.4)
		**Period effect**	–	–	−0.9 (2.3); 95% CI: −1.7, − 0.1
					*P* = 0.475
Linear-fit Solid T_1/2_	T_1/2_ (min.)	**Group 1**	114.5 (10.6)	113.8 (9.9)	0.8 (5.6)
		**Group 2**	105.2 (6.7)	102.2 (6.0)	−3.0 (9.3)
		**Period effect**	–	–	−0.8 (7.5); 95% CI: − 3.6, 1.9
					*P* = 0.307

Data are presented as mean (SD)

Group 1: ^99m^Tc-SC, then ^99m^Tc-SP (*n* = 18)

Group 2: ^99m^Tc-SP, then ^99m^Tc-SC (*n* = 13)

**Fig. 5:**
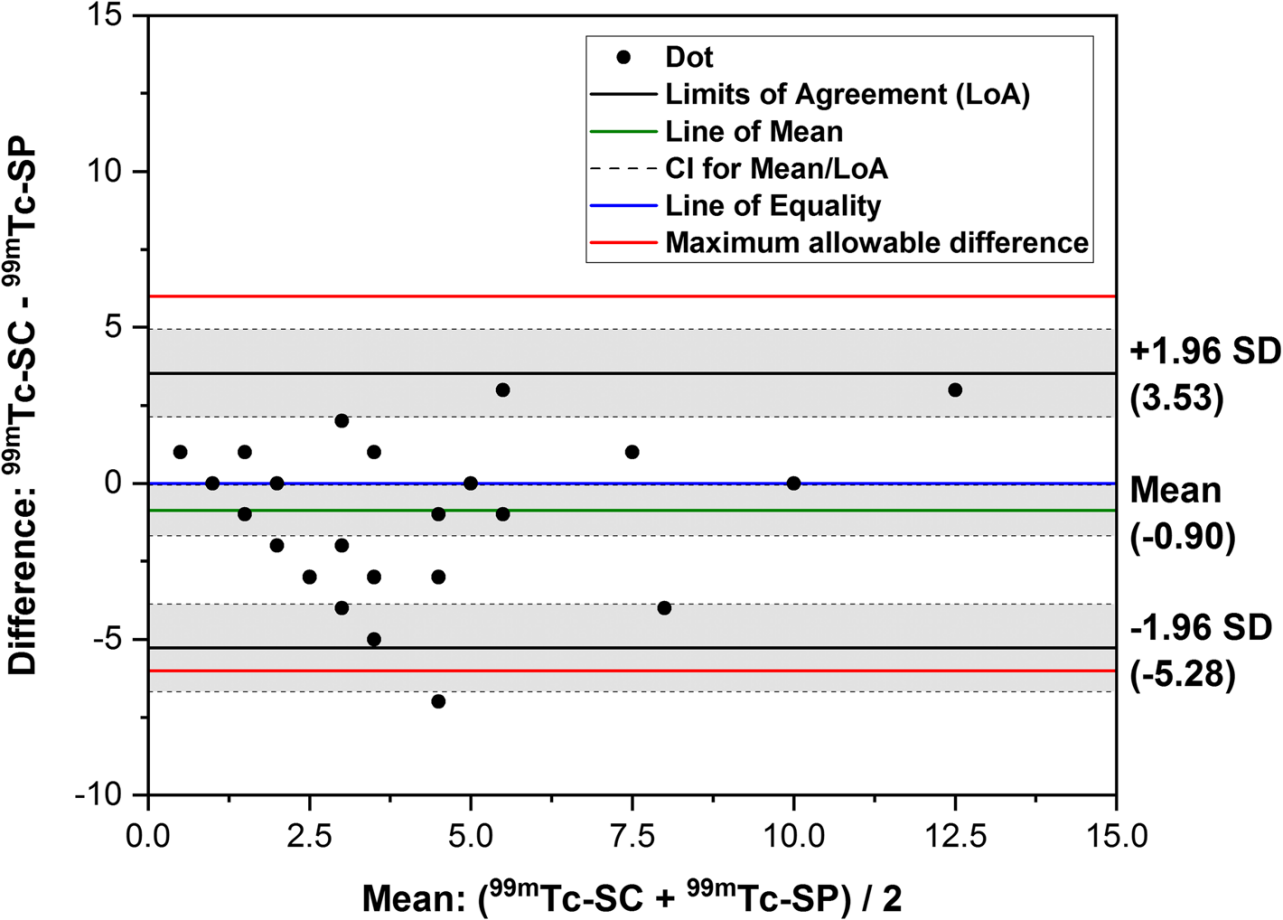
Bland-Altman graph showing the difference of total gastric meal retention (GMR) between ^99m^Tc-SC and ^99m^Tc-SP the 4th-hour time frame, plotted against the mean measurements. The analysis showed a good agreement between the two radiotracers, and both limits of agreement fell within the defined maximum allowable difference of ±6%

According to the B-A plot analysis (Fig. 5), it could be noted that the average difference of GMR percentages between the two radiotracers, as an estimate of the agreement, is considerably small, where on average, the measured GMR for ^99m^Tc-SC is 0.9% ± 2.3% lower than ^99m^Tc-SP at the 4th-hour mark. Almost all the dots (96.8%; 30/31) fell within the narrow LoAs (and the 95% CI indicated by the shaded regions) and decently clustered around the line of equality. The LoAs were also found to be within the acceptable 6% maximum allowable difference. Furthermore, there is a moderate positive correlation (Kendall’s Tau (τ) coefficient = 0.498, *P* < 0.001) between the 4th-hour time-point GMRs for both ^99m^Tc-SC and ^99m^Tc-SP.

## Discussion

Using the standardized egg-based meal, our results showed that the GMRs for ^99m^Tc-SP and ^99m^Tc-SC are similar. The calculated normative values for each time point for ^99m^Tc-SP are in agreement with the universally-accepted upper normal limits, i.e., 90% at 1 h, 60% at 2 h, 30% at 3 h and 10% at 4 h, and these GMR values commensurate with previous reports on ^99m^Tc-SC [5]. We have also reported the normal values for gastric emptying T_1/2_, and similar to GMRs, no significant differences were found between the results for ^99m^Tc-SP and ^99m^Tc-SC.

The main strength of this prospective imaging study is the cross-over design, which minimized the noise from inter-subject variations and reduced the effect of small-sample statistics (fewer participants are required to achieve the same precision as a traditional parallel-group design) [18]. However, even with the intended equal randomization, an optimal within-individual comparison could not be made due to the unbalanced group sizes (5 participants from Group 2 were excluded in the final analysis). This flaw might introduce a power issue, which could hamper the study’s ability to detect and adjust for a period effect. It is clear from the outset that there are no prior reasons to anticipate a period effect as it is assumed that the healthy participants are stable over time, and they do not have any underlying chronic medical conditions. The two-week interval was also relatively short and deemed adequate to observe similar gastric emptying patterns. Nonetheless, the analysis for the period effect conducted on the available dataset showed statistically insignificant results across all of the GES parameters. Hence, it is estimated that this methodological disadvantage does not have a substantive effect on the results presented here.

The B-A plot analysis has shown that both radiotracers have a small systematic difference only, where ^99m^Tc-SP provided a slightly higher average GMRs compared to ^99m^Tc-SC. Additionally, the good agreement levels and correlations between the two radiotracers further support ^99m^Tc-SP as a useful alternative to ^99m^Tc-SC. In the absence of any previous trials comparing the two radiotracers, this study provided valid evidence that ^99m^Tc-SP is as good as ^99m^Tc-SC and comes at a cheaper cost.

Although considered as the gold-standard, only a handful of nuclear medicine centers around the world that can perform routine GES due to the high price of ^99m^Tc-SC kits. In this regard, a standard Technephyte kit with five sterile vials is much more cost-effective at MYR 920 (USD 220) in comparison to the price of the Pharmalucence kit with the same number of vials, which could reach up to MYR 4860 (USD 1161). This pricing gap indicates that the use of ^99m^Tc-SP could generate an almost 5-folds net saving of MYR 3940 (USD 940), and will be especially useful in low resource settings, i.e., in LMICs. Other advantages of Technephyte kits include not needing any boiling procedure, which simplifies the radiotracer preparation and minimizes the radiation exposure to staff members inside a hot laboratory. On top of that, any readily-available extra doses from GES can be immediately dispensed to perform other colloid-based scintigraphy procedures, i.e., lymph node mapping and liver-spleen imaging [19, 20].

A potential drawback that could arise with the use of ^99m^Tc-SP is the small colloidal size (particle mean size: 50–150 nm), which may easily evaporate into the air during the cooking of egg-whites compared to the larger ^99m^Tc-SC (particle mean size: 61–445 nm). This limitation could cause an unsatisfactory radiolabeling efficiency of the egg-white protein (ovalbumin) and thus affecting the test meal’s overall stability inside the human gastric fluid. In the present study, the similar gastric emptying patterns shown by both radiotracers were indirectly an indication of comparable radiolabeling stability. The reason for this may be due to the adding of radiotracers to the egg-white pre-cooking, which is compliant to the full consensus protocol. This method allows better affixation of the radiocolloid particles to the ovalbumin as the egg-white denatures, coagulates, and solidifies during the cooking process. Comparatively, adding the radiotracer during or post-cooking can reduce the radiolabeling stability and at risk of creating false-negative GES [21]. Misdiagnoses due to improperly performed GES would have a significant impact on patients’ quality of life, erode physicians’ confidence in diagnosing, and increase the burden of the healthcare system [22]. Therefore, with the data at hand, our findings suggest that the protocol ensures excellent radiolabeling stability and in vivo performance of gastric emptying parameters, at least for ^99m^Tc-SP and ^99m^Tc-SC.

To exclude the potential confounding effect of sex on gastric emptying [23, 24], we have performed a separate male and female subgroup analyses. Both sexes showed no significant differences in GMRs and T_1/2_ between ^99m^Tc-SC and ^99m^Tc-SP (all *P* > 0.05; Additional file 1). The individual data measured for all participants at the 4th-hour, regardless of sex, are well below the international GMR reference value of 10% – except for one female (^99m^Tc-SP: 11%, ^99m^Tc-SC: 14%). Upon review of our information sheet, it was found that the particular participant underwent both GES sessions during the luteal phase of her menstrual cycle. Hormonal effects have been proposed as a possible reason for delayed gastric emptying among premenopausal women – citing elevated levels of estrogen and progesterone during the luteal phase may inhibit smooth GI muscle activity compared to the follicular phase [25, 26]. At the moment, we are unable to appropriately compare the two phases for both radiotracers because of the limited subgroup population (see footnote a; Additional file 1). The slight emptying delay, however, remains within the pre-defined normal cut-off range (< 16% at 4th-hour), and any differences between both radiotracer measurements are unlikely to be of clinical significance.

## Conclusion

In conclusion, this study shows that ^99m^Tc-SP is a reliable alternative radiotracer to ^99m^Tc-SC for GES. This study has also established the normative data of both radiotracers in GES for the healthy multi-ethnic Asian population.

## Supplementary information

**Additional file 1: Table S1.** Separate comparison of gastric emptying parameters according to sex subgroups.

## Data Availability

The minimal data that supports the conclusions of this study are included in the article. The full datasets are not publicly available due to de-identified data sharing restrictions by the Human Research Ethics Committee of Universiti Sains Malaysia. The reason is that the data contain sensitive information (hospital registration number) and are easily identifiable as they come from one single hospital. Data are, however, available from the corresponding author upon reasonable request and with permission of the Human Research Ethics Committee of Universiti Sains Malaysia.

## Abbreviations

^99m^Tc-SP: ^99m^Tc-sodium phytate
^99m^Tc-SC: ^99m^Tc-sulfur colloid
GES: Gastric emptying scintigraphy
ARSAC: Administration of Radioactive Substances Advisory Committee
^99m^Tc: Technetium 99-metastable
GI: Gastrointestinal
SNMMI: Society of Nuclear Medicine and Molecular Imaging
ANMS: American Neurogastroenterology and Motility Society
FDA: Food and Drug Administration
LMIC: Low- and middle-income countries
ROI: Region-of-interest
GMR: Gastric meal retention
T_1/2_: Half-emptying time
IQR: Interquartile range
SD: Standard deviation
CI: Confidence interval
CLSI: Clinical & Laboratory Standards Institute
B-A: Bland-Altman
LoA: Line of agreement
BMI: Body mass index
