# Supplementary material for: 99mTc-sodium phytate is a valid alternative to the gold-standard 99mTc-sulfur colloid in the measurement of gastric emptying among healthy multi-ethnic Asian population: results of a randomized cross-over trial
Source: BMC Gastroenterol. 2020 Aug 31;20:293. doi: 10.1186/s12876-020-01426-5 (PMC7457507; doi:10.1186/s12876-020-01426-5)
Supplement: Supplementary file 1 — Additional file 1: Table S1. Separate comparison of gastric emptying parameters according to sex subgroups. [file 12876_2020_1426_MOESM1_ESM.docx]

| **Parameters** | | **Males (*n* = 17)** | | ***P* ^b^** | **Females (*n* = 14)^a^** | | ***P* ^b^** |
| --- | --- | --- | --- | --- | --- | --- | --- |
|  |  | **^99m^Tc-SC** | **^99m^Tc-SP** |  | **^99m^Tc-SC** | **^99m^Tc-SP** |  |
| Total gastric meal retention | 0.5 hr. (%) | 77.0 (12.0; 93.3) | 86.0 (20.0; 100.0) | 0.053 | 84.0 (8.0; 94.7) | 84.0 (7.0; 90.5) | 0.855 |
|  | 1 hr. (%) | 55.0 (26.0; 82.5) | 67.0 (32.0; 92.7) | 0.088 | 71.5 (23.0; 91.2) | 72.5 (16.0; 84.6) | 0.987 |
|  | 2 hr. (%) | 16.0 (17.0; 39.6) | 19.0 (26.0; 50.4) | 0.347 | 48.0 (33.0; 72.4) | 44.0 (19.0; 58.4) | 0.533 |
|  | 3 hr. (%) | 4.0 (3.0; 8.36) | 4.0 (5.0; 17.34) | 0.455 | 12.0 (21.0; 35.5) | 16.5 (9.0; 30.0) | 0.702 |
|  | 4 hr. (%)^c^ | 1.0 (1.0; 4.05) | 2.0 (2.0; 7.31) | 0.101 | 4.5 (5; 13.1) | 5.5 (3; 11.3) | 0.258 |
| Linear-fit  Solid T_1/2_ | T_1/2_ (min.) | 103.4 (5.0; 111.7) | 100.1 (8.0; 116.5) | 0.579 | 117.7 (18.7; 135.3) | 116.6 (13.0; 132.5) | 0.808 |
| Data presented as median (IQR; 95^th^ percentile of smoothed empirical likelihood quantile).  ^a^Menstrual cycle subgroup population (similar for both GES sessions): Follicular phase (*n* = 5); Luteal phase (*n* = 6); Unknown (*n* = 3).  ^b^No significant differences in the median percentages of all parameters between ^99m^Tc-SC and ^99m^Tc-SP for both sexes (*P* > 0.05).  ^c^GMR percentage at the 4^th^-hour was modified according to DiBaise’s stratification: normal < 16%, abnormal ≥ 16% [5, 16]. | | | | | | | |

**Additional file 1**

Supplementary table 1: Separate comparison of gastric emptying parameters according to sex subgroups.
